# Effect of Absorptivity of Superabsorbent Polymers on Design of Cement Mortars

**DOI:** 10.3390/ma13235503

**Published:** 2020-12-02

**Authors:** Jan Fořt, Przemysław Migas, Robert Černý

**Affiliations:** 1 Faculty of Civil Engineering, Czech Technical University in Prague, Thákurova 7, 16629 Prague, Czech Republic; przemyslaw.migas@pk.edu.pl (P.M.); cerny@fsv.cvut.cz (R.Č.); 2 Faculty of Chemical Engineering and Technology, Cracow University of Technology, Warszawska 24, 31155 Cracow, Poland

**Keywords:** superabsorbent polymer (SAP), absorptivity, teabag method, filtration method, mechanical performance, flow table test

## Abstract

The functional properties of composites modified by superabsorbent polymers (SAPs) strongly depend on the swelling capacity of applied SAPs. In this sense, three types of commercially available SAPs namely Cablock CT, Hydropam, and Creasorb SIS with different chemical composition and particle size distribution were studied in this manuscript to reveal the differences in absorptivity as can be viewed as pretests for their utilization in concrete composites. In addition, absorptivity in distilled water, tap water, and 0.1 M NaCl solution are examined for determining the SAPs response for the change of the solution pH. To overcome problems with the teabag method inaccuracy, the new method is introduced. Besides to quantitative evaluation of the SAPs absorptivity, the correlation for the absorption and desorption period as the function of SAPs residence time within the examined solution is proposed. To access the effect of selected SAPs on functional properties, optimization based on the flow table test is employed and mechanical parameters are determined after 7, 14, 28, and 90 days of curing. Obtained results refer to substantial differences between particular SAPs and contribute to the understanding of the effect of SAP on the functional properties of cement-based materials.

## 1. Introduction

Superabsorbent polymers (SAPs) are formed by hydrophilic networks that have to allow absorb, retain, and release (under changes in their environment), a significant amount of water or aqueous solution [1]. SAPs due to their unique properties are used in a plethora of applications, like hygiene and medical purposes [2,3,4,5], and as nutrient carriers, and water reservoirs applied in agriculture [6,7,8]. Another useful application can be found in concrete materials, where a number of possibilities are available due to their ability to control (absorption/release) free water in the cement-based mixture [9,10,11]. In this sense, SAPs have attracted increasing attention during the last decade due to the capability to mitigate drying shrinkage and promote self-healing in high-performance constructions [12]. Here, SAPs serve as small water reservoirs evenly distributed across the concrete volume, which can slowly release water during the cement hydration progress, and maintain optimal internal humidity during the curing stage or alternatively supply water to unreacted cement particles to boost self-healing [13]. Further water penetration from the environment may also contribute to a higher level of cement hydration [14,15]. Application of SAPs in concrete provides also improvement in the freeze/thaw resistance due to the formation of fine voids that play an important role in preventing internal pressure during water freezing [16].

Such modified materials show highly improved properties, by the meaning of mechanical strength, ductility, and durability [17]. Unfortunately, inaccurately selected dosages of SAPs taking into account the chemical composition and particle size may result in the worsening of functional properties of SAP modified cementitious materials [18]. On this account several research papers aimed at investigating the correlation between the chemical structure of SAP and the applicability in concrete mixtures as internal curing agents. Namely, the work of Tan et al. [19] discussed the dosing methods applied to support the beneficial effect of SAPs. As revealed, the addition of preswelled SAP led to a noticeable decrease in the compressive strength compared to the application of the powdered SAP. The variety of dosing methods were studied by Woyciechowski and Kalinowski [20], who contrary to the previously described study found the application of preswelled SAP as more efficient taking into account the preservation of mechanical performance. Since the swelling rate and capacity have been found as a crucial parameter for precise concrete design, the work of De Meyst et al. [14] highlighted the importance of the cross-linking degree of the SAPs. As noticed, the amount of additional water needed to obtain the same workability level of the mixture must be determined for each SAP material to avoid potential problems driven by a high swelling rate, hydration delay, and consequent deterioration of functional properties. The synergic effect of the application of SAPs and fly ash confirmed that Yang et al. [21] mentioned the synergistic effect of multiple ions. A more detailed elucidation of SAPs incorporation in cementitious materials can be found in the work of Mechtcherine et al. [22] who investigated the effect of molecular structure and applied solution on the autogenous shrinkage and mechanical strength of concrete mixtures. Considering the obtained findings, one can distinguish a correlation between SAP swelling and the ionic composition of applied liquids. Authors refer to a substantial influence of anion density on absorption and desorption behavior that closely correlates with the rheology of the fresh mixture [23]. Additionally, the kinetics of pore solution uptake and release during the concrete curing and maturing period in order to mitigate the autogenous shrinkage. On the other hand, the crosslinking density, as one of the parameters used for SAP description was not concluded as important for SAPs behavior. The research paper focused on the elucidation of the principles of SAPs application in cementitious materials with emphasis on the final strength in the hardened state based on the approximation of the Powers model [24,25]. The provided conclusions refer to the possible mitigation of high-performance concrete self-desiccation by the help of SAPs as agents for the formation of the desired pore distribution that promotes optimal curing conditions. On top of that, the frost resistance of such modified mixtures can be improved due to gas-filled voids produced during mixture preparation [26].

Mignon et al. [27] compared the self-healing capacity and compressive strength of obtained mortars. The research provides important information related to the application of commercial SAPs, and other types like modified methacrylate alginate combined with acrylic acid, and acrylamide, and methacrylate chitosan combined with the monomer dimethyl aminopropyl methacrylamide.

Different SAP samples behave individually in cement-based materials depending on their chemical structure like cross-linking and functional group density and depending on their particle size distribution. Hence the proper use of SAP materials in cement mixtures requires knowledge of their basic properties. SAP absorptivity is one of the most important factors that characterized this type of material in terms of the efficient design of concrete composites. It translates into the rheological properties of the fresh concrete paste, which in turn is important in terms of its homogenization and pumping [28]. Understanding of the SAP absorption characteristic allows for proper dosage of additional water, which in addition to the aforementioned rheological properties, also translates into the mechanical properties of resulting concrete. This work presents the absorptivity of commercially available SAPs depending on the time, pH, and type of used solution. The presented results are the starting point for further research of SAPs application in cement-based materials.

## 2. Materials and Methods

### 2.1. Materials

**Figure 1:**
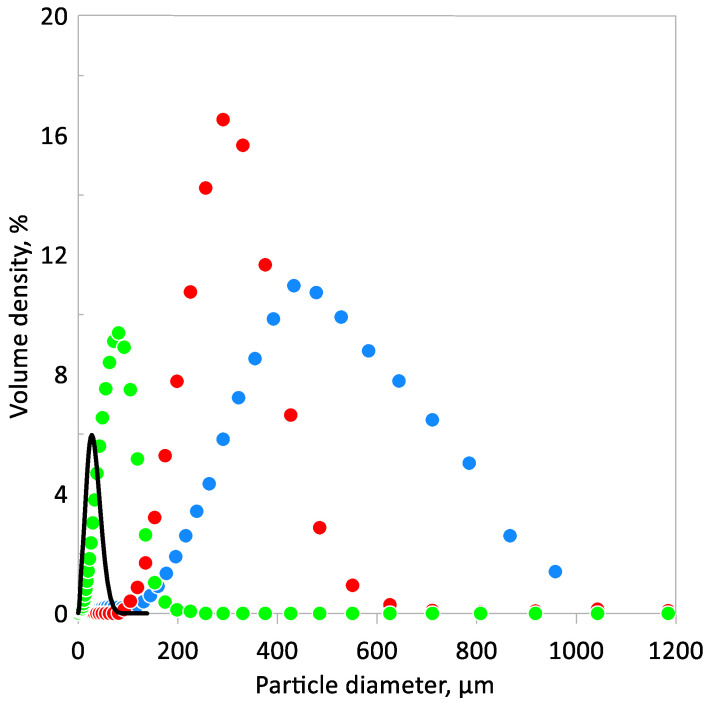
Particle size distribution of superabsorbent polymer (SAP) ● Cablock CT, ● Hydropam, and ● Creasorb SIS—Cement.

Three SAPs with different particle size and chemical composition, namely Cabloc CT, Creasorb SIS, and Hydropam (all obtained from Evonik Industries Ltd., Essen, Germany), were examined. All tested polymers are considered as non-degradable and non-toxic materials [29]. Cabloc is based on cross-linked sodium polyacrylate, Creasorb on cross-linked acrylamide/acrylic acid potassium salt, and Hydropam on sodium salt and acrylamide/acrylic acid copolymer. Used SAPs differ mainly in terms of solubility, while Cabloc and Creasorb do not dissolve in water, Hydropam is slightly soluble and creates a gel. The next difference between SAPs is the particle size distribution, which was analyzed by the laser particle size analyzer Bettersizer S3 Plus working on the laser diffraction principle with a measuring range from 0.01 to 3500 μm. The particle size distributions of the used SAPs are presented in Figure 1. One can observe quite scatter results between SAPs. Hydropam is characterized by a wide range of distribution and the largest particle sizes. In contrast, the Creasorb has the lowest particle sizes and narrowest range of results. The highest volume fractions are for particles diameters of 81, 291, and 433 μm for Creasorb, Cablock, and Hydropam respectively, while mean particles sizes *d_M_* are 63 μm for Creasorb, 291 μm for Cablock, and 526 μm for Hydropam. The mean particle diameter is expressed as:

(1) $$d_{M}=\overset{n}{\underset{i=1}{\sum }}\left(V_{i}\;\cdot \;d_{i}\right)$$

where *V_i_* is the volume density and *d_i_* the particle diameter.

### 2.2. Absorption Experiments Procedure

**Figure 2:**
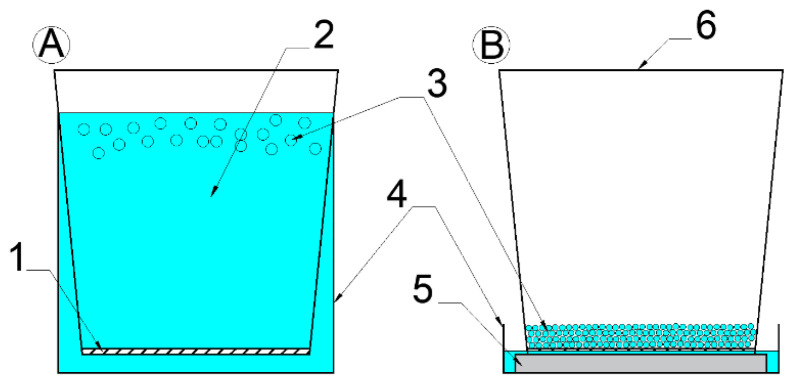
Scheme of the absorption experiment (**A**) conventional experiment; (**B**) modified experiment for Creasorb: 1. filtration cloth, 2. absorbed liquid, 3. swelling SAP, 4. vessel with solution, 5. sponge, and 6. vessel with filtration cloth (VFC).

Research papers aimed at the determination of SAPs absorption capacity, as accessed in [18,21,30,31], refer to the correlation between the solution applied and SAPs composition. The teabag method has been found as the most employed method for this purpose. However, this method has several limitations due to the formation of a large amount of interparticle liquid that may result in substantial overestimation of absorption results. In this sense, absorption experiments for Hydropam and Cablock materials were carried out in a way that is based on the filtration and teabag methods [32]. There was used the vessel with the examined solution contained filtration cloth at its bottom—vessel with filtration cloth (VFC), the proposed equipment is a kind of synthesis of the above-mentioned methods (the scheme is presented in Figure 2A). The same vessel was used in the study of Creasorb material (Figure 2B), except that unlike previous SAPs, this vessel was not immersed in the solution, but only its bottom was wetted. This method, in turn, is based on the AUL (absorbency under load) examination proposed by Zohuriaan [8]

Absorption process studies were conducted at 20 °C [28] (the small temperature fluctuations would not have a significant impact on the process). Initially, the vessel with filtration cloth was wetted in the tested liquid, and before weighing its outer surface was wiped with a paper tissue. In Cablock and Hydropam sorption studies, time measurements were started when the SAP (Cablock *m*_0_ = 100 mg, Hydropam *m*_0_ = 300 mg) were introduced to the vessel with filtration cloth (immersed in the beaker with examined liquid). After reaching a certain time, the VFC was lifted, allowing excess liquid to drain. In the case of Cablock, additional filtration under reduced pressure in the Buchner funnel was used, in order to get rid of excess fluid that could overestimate the real SAP absorptivity [33]. No vacuum was applied to the Hydropam, which is associated with its gelation, and the use of a vacuum would cause the penetration of the formed gel to the filtered liquid. Due to slight Hydropam solubility, the standard teabag method caused the gel release into the surrounding solution. In the method presented here, the Hydropam absorption proceeded in the upper surface of the solution (Hydropam settlement was possible provided that this SAP was gently introduced to the examined liquid). After lifting the VFC, the solution drained, and the swelled Hydropam settled on the filtration cloth. Slight gel leakage from the VFC bottom was observed after a long time, i.e., 1 h, which may potentially underestimate the absorption results. The sorption studies for Creasorb were carried out only for 1 h. This is due to the relatively low particle sizes and internal resistance of fluid permeability between particles. As a result, at the beginning of the process, only part of the material was in the solution scope, so an additional plastic stick was used (weighed before and after the experiment) in order to gently mix the SAP with the absorbed solution. In these tests, 300 mg of Creasorb was added to the VFC, and after placing it on a damp sponge, the time measurement was started. In the case of this SAP, it was not possible to use the teabag or filtration method, because introducing Creasorb to an excessive amount of liquid (like in the previous SAPs) would form the composition with swelled SAP and a large amount of interparticle liquid, which would overestimate the absorption results. Kang et al. [33] used centrifugation to get rid of the excess solution. However, in his work, SAPs with a diameter of over 100 μm were examined thus the method would rather not be translated for such small particles. In all cases after a certain time, the VFC was lifted to let the liquid drain, and its outer surface (especially filtration cloth) was wiped off excess solution with a paper tissue, next to the whole (vessel and swelled SAP) was weighed. The sorption process experiments were repeated at least three times for a given sample and a given time.

Six types of liquid were used in sorption experiments: distilled water (pH = 6.13), tap water (pH = 7.61), 0.1 NaCl (pH = 6.40), and three cement based solutions 2.5 w/c (pH = 12.64), 5 w/c (pH = 12.57), and 10 w/c (pH = 12.46), where w/c means the distilled water to cement mass ratio. pH was measured with a PC 70+DHS portable pH-meter (PC70 vio DHC, XS instruments, Italy). All the cement-based solutions were filtered before use with the filtration paper to remove particles larger than 5 μm.

The absorptivity (*A*) of the SAP is expressed as the ratio between mass after certain sorption time *m*_1_ and the initial mass of the sample *m*_0_.

(2) $$A=\frac{m_{1}}{m_{0}}$$

To evaluate the absorptivity against elapsed time (*τ*), the following equation was proposed (based on [34])

(3) $$A=A_{M}\cdot (1-a\cdot e^{b\cdot \tau })$$

where *A_M_* is the maximum absorptivity value while *a* and *b* are the characteristic coefficients for the SAP-solution system. To derive the *a*, *b* values, the above equation has been transformed into a linear form, where ln(*a*) is the intercept and b is a function slope.

(4) $$\ln\left(1-\frac{A}{A_{M}}\right)=\ln\left(a\right)+b\tau$$

The presented equation was used to describe the absorption and desorption periods of SAPs.

### 2.3. Determination of Extra Water Dosage

To reveal the optimal ratio between applied mixture constituents and keep the similar rheology properties of the fresh mixture, the flow table test was used according to EN 12350-5 [35]. The amount of extra water was modified in 5 wt.% steps (by the mass of the initial water dosage) for each mixture with SAP content 0.6, 0.8, and 1.0 wt.% respectively to reach the optimal flow spread as obtained for the reference mixture. The flow table test of designed mortars was determined according to standard EN 12350-5, in this sense, the measured spread diameter was approximately 160 mm in both perpendicular directions. Initial mixture proportions are given in Table 1. Where C, H, and S mean concrete mixture with Cablock CT, Hydropam, and Creasorb SIS addition and denotation of SAP dosage as follows: 1–0.5 wt.%, 2–1 wt.%, and 3–1.5 wt.%. The extra water dosage (ew/c) was determined according to the preliminary results from the mixture design based on adverse effects on rheologic properties. For the preparation of cement mortars, ordinary Portland cement (Cemex, Ltd., Prague, Czech Republic) composed of 62.69% CaO, 15.84% SiO_2_, 4.57 Al_2_O_3_, 2.33% Fe_2_O_3_, 2.64% MgO, 2.93% SO_3_, 0.63% K_2_O, and 0.25 NaO and specific density of 3090 kg/m^3^ was used. Sand in fraction 0/4 mm was used.

Prepared samples were stored for 28 days at the highly humid environment at 21 °C. All experiments were performed under laboratory conditions (21 °C/35% RH).

The mechanical strength by the meaning of compressive strength and flexural strength were determined after 28 days of curing by VEB WPM Leipzig (Markkleenberg, Germany) having stiff loading frame with a capacity of 3000 kN. Both tests were carried out according to Standard EN 1015-11 [36]. The flexural strength was 170 measured in a three-point arrangement on three prismatic samples with dimensions of 40 mm × 40 mm × 160 mm. The rest of the broken prisms were used for the compressive strength experiment.

## 3. Results and Discussion

### 3.1. Absorptivity Results

**Figure 3:**
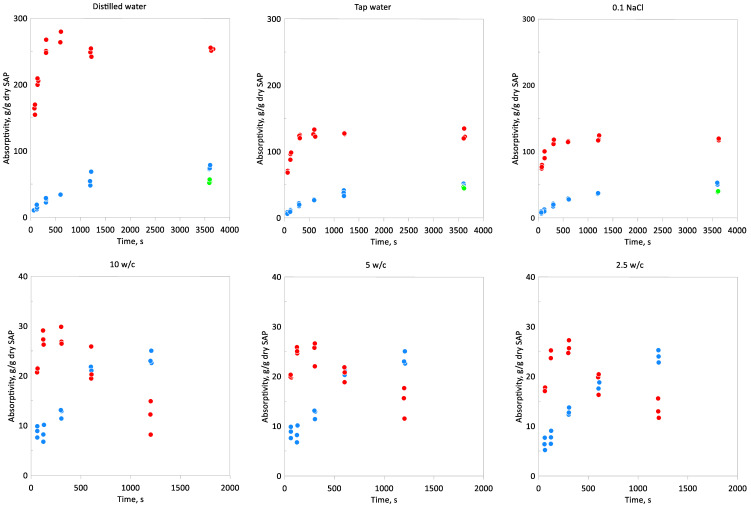
Results of sorption experiments as a time function: ● Cablock CT, ● Hydropam, and ● Creasorb SIS.

The results of the sorption process are presented in Figure 3. The highest *A* value was observed for the distilled water, the absorptivity after 1 h was about 250 for Cablock, 75 for Hydropam, and 55 for Creasorb. The swelling capacity decreased in the tap water and the sodium chloride solution, but the difference in that the factor between those two solutions was rather low. For Cablock in the tap water and 0.1 M NaCl solutions, the swelling capacity (1 h) was about 2 times lower than for the distilled water, in the case of two other SAPs it does not change so radically. This relation is even more pronounced in the cement-based solutions where Cablock absorptivity after 1 h reached values below 10. This indicates a higher sensitivity on ions concentration for Cablock superabsorbent polymer. For Cablock in the water and NaCl solution, the equilibrium state is reached relatively fast, in the 5th minute of the experiment, which is a big difference relative to Hydropam. In the cement-based solutions, the initial Cablock absorption is also faster. A relatively fast velocity of swelling can be explained by smaller grain sizes, compared to Hydropam. This observation is consistent with results obtained by Yun [37] where the highest initial absorption kinetic for the finest SAP particles was observed. It is noteworthy that authors also noticed lower absorptivity at the equilibrium state for smaller mean particle diameter. The fastest sorption was visually observed for Creasorb, due to the smallest grain sizes, but it was also accompanied by the internal resistance of fluid transportation between particles. As mentioned in the fifth minute of the experiment, for the water and NaCl solution, the Cablock absorptivity reaches equilibrium values. This characteristic was completely different in the case of cement-based solutions. For these solutions, the clear sorption maximum was observed for this time, followed by the desorption step and weight loss of swelled SAP. The desorption stage was characterized by a lower rate in comparison to the absorption period. This behavior indicates that in the real cement paste the initial absorption would increase the viscosity of the fresh concrete mixture, which should be compensated over time by the following desorption process. Similar characteristics have been observed, among others [29,31], where authors also based studies on sodium polyacrylate, one difference is that in our case the absorptivity after 1 h of experiment reaches much lower values. In addition to raw cement-based solutions, Kang et al. [33] tested compensation of Na^+^ and K^+^ ions, to prepare the cement-based solution with more realistic ions concentration and to better reflect the environment in the fresh concrete paste. He stated that additional compensation caused mitigation of the desorption period. This information potentially shed new light on the Cablock influence on the cement paste workability, and the formation of the micro void in the prepared concrete and should be taken into account in further research. Surprisingly there were no significant differences between examined cement-based solutions, regarding the swelling capacity. Absorptivity results for Hydropam and Creasorb were very similar in all tested cement-based solutions. Only one observation was noticed, namely a slightly higher rate of swelling for Cablock in the first 5 min with an increasing w/c ratio. In all tested solutions, the absorptivity of Hydropam gradually increased with elapsed time, the highest value was achieved for the distilled water, the lowest for cement-based solutions. As was mentioned the Hydropam absorptivity after 1 h was above 70 in the distilled water, in turn in the cement-based solution its value was about 30. Final A values for the Creasorb were about 55 and 30 for the distilled water and the cement-based solutions respectively.

**Figure 4:**
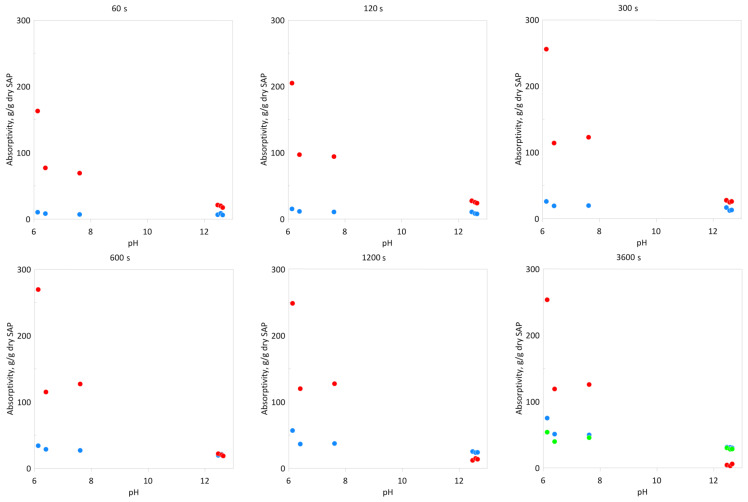
Absorptivity of superabsorbent polymers depending on time and pH of the solution: ● Cablock CT, ● Hydropam, and ● Creasorb SIS.

In Figure 4 absorptivity as the function of a pH of the solution applied is presented, where elapsed time is a parameter and presented points are mean values of previous absorptivity tests. A decrease in the absorptivity with increasing pH was observed, where the Cablock had the highest sensitivity to a change of solution pH, while Creasorb had the lowest. For example, after 1 h absorptivity for Cablock (in the examined pH range) differed about 50 times, while for Creasorb it changed less than 2 times. Mignon [38] noticed the increase of swelling capacity for acrylic acid and acrylamide based super absorbent polymers in pH = 12, which was caused by amide groups hydrolysis [39]. In this case pH of the solution was adjusted by NaOH addition, so these results do not exactly correlate with our tests, because of Ca^2+^ presence in cement-based solutions, which causes a screening effect and swelling reduction [33]. Mignon et al. [40] also checked the swelling response on pH change in the acidified cement-based solution (pH < 13), and a similar rise of the absorptivity was not observed. The effect of hydrolysis may explain the lower sensitivity to pH changes for acrylic acid and acrylamide based (Hydropam and Creasorb) SAP compared to Cablock.

Taking into account the effect of different particle sizes, the water absorption capability of SAPs depends on the diameter of particular SAP. As can be seen, SAPs with smaller diameter exhibited faster and more intense swelling compared to larger particles. This fact complies with the findings of He et al. [41]. On the other hand, the smaller particles are more prone to agglomeration, thus advanced homogenization techniques are required for the preparation of concrete mixtures [42]. In this sense, the particle size has an important influence on the strength parameters as reported by Ma et al. [43]. Such results are in contrast with the investigation of De Meyst et al. [44], who did not reveal any notable effect on mortar properties related to the particle size of studied SAPs.

Table 2 presents the values of the ln(*a*), *b* coefficients for the solution–SAP system (based on Equation (4)), for the absorption (*S*) and desorption (*D*) period. Hydropam was characterized by constant absorption in the whole tested time, while for the Cablock we could distinguish two periods; the first 5 min (*S* period to achieve equilibrium or max swelling capacity), and the desorption step. The model presented here, describes the Hydropam swelling capacity in the absorption period in a much better way than for Cablock. In that case, it is characterized by higher inaccuracy, especially in cement-based solutions. In addition, in liquids, slightly better results for the Cablock desorption were obtained than for the absorption step. The introduced model described the Hydropam swelling capacity in the absorption period in a much better way than for Cablock. R^2^ for Hydropam sorption in all tested solutions was higher than 0.9. In the case of Cablock, higher inaccuracy could be observed, especially in cement-based solutions where R^2^ was even lower than 0.2. In addition, in liquid slightly better results (R^2^ about 0.6) for the Cablock desorption were obtained than for the absorption step. The possible explanation can be found in the fact that the significantly worsened results for Cablock in cement-based solutions are based on two parallel processes and not only sorption. Water sorption and its delayed desorption caused by slower diffusion of Ca^2+^.

### 3.2. Optimization of Water Dosage

**Figure 5:**
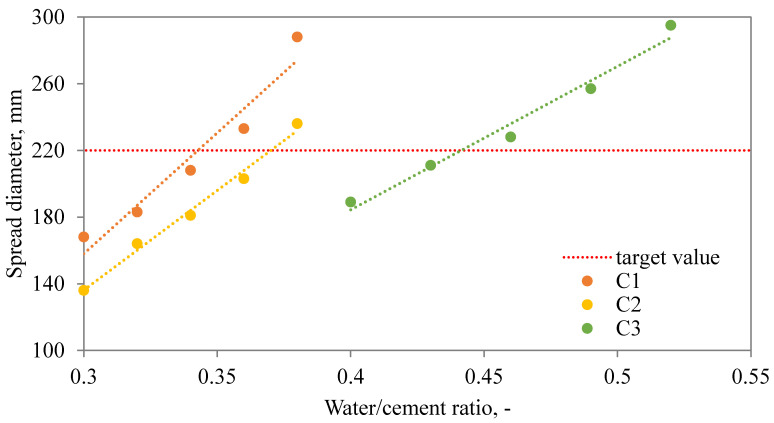
Dependency between spread diameter and additional water dosage for C mixtures.

**Figure 6:**
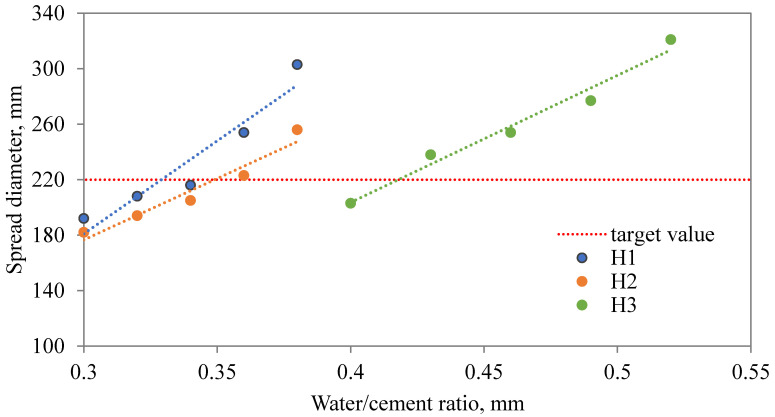
Dependency between spread diameter and additional water dosage for H mixtures.

**Figure 7:**
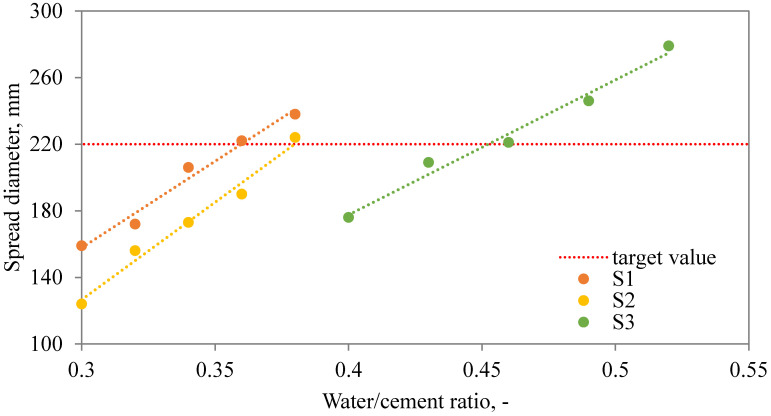
Dependency between spread diameter and additional water dosage for S mixtures.

The application of SAPs in cementitious composites substantially affects the amount of water needed for the sufficient workability level of the mixture. The extra water dosage relies on many parameters related to the SAP characteristics such as chemical composition, diameter, SAP, dosage, etc. In order to access the optimal ratio for each SAP studied within our research, the effect of extra water dosage on the flow table spread diameter was studied. As plotted in Figure 5, Figure 6 and Figure 7, the relationship between the extra water added and spread diameter as to be distinguished according to the amount of SAP applied.

As one can see, the relationship between the spread diameter and extra water added can be described and optimal extra water dosage estimated. Considering the obtained results, the optimized water dosage was given in Table 2. Revealed results point to a diverse behavior of studied SAPs. While Hydropam did not affect the rheology of the fresh mixture to a great extent, the other two SAP types induced substantial changes and require a higher water dosage. To be more specific, the H1 mixture needs to be supplemented by only 0.03 extra water, i.e., 0.32 in total, mixture C1 requires 0.345 and S1 even 0.36, for mixtures with 0.6 wt.% of SAP applied. Similar results can be observed for higher SAP admixture ratios also. Revealed findings refer to the importance of the detailed characterization of used SAP admixture and its applicability in cement composites with emphasis on rheologic properties. Considering the present state-of-the-art, the characterization of SAP mixing methods need to be described together with the limitations given by the SAP properties [14,41,45]. In other words, the effect of SAP on plastic viscosity and cement mixture workability poses a complex issue including the dependency of SAP swelling on the concentration of selected ions in the cement solution [18].

Based on the obtained results from flow table test experiments, the mixtures with optimal w/c ratio were redefined as given in Table 3.

The casted samples having dimensions of 40 mm × 40 mm × 160 mm were stored for 3, 7, 28, and 90 days in water at 21 °C and consequently used for determination of the strength parameters by the meaning of compressive and flexural strength.

### 3.3. Mechanical Strength

**Figure 8:**
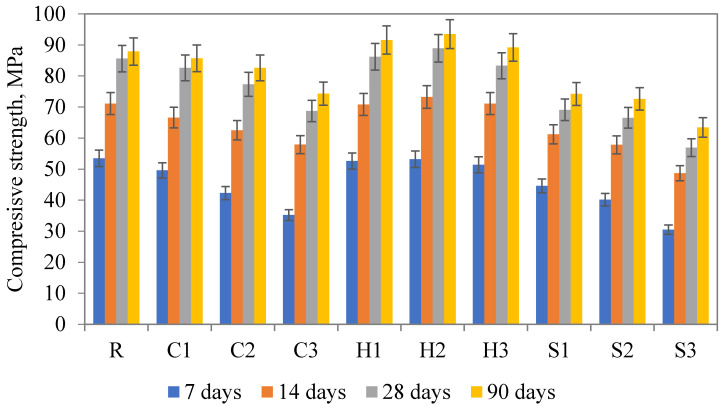
Compressive strength of studied materials.

**Figure 9:**
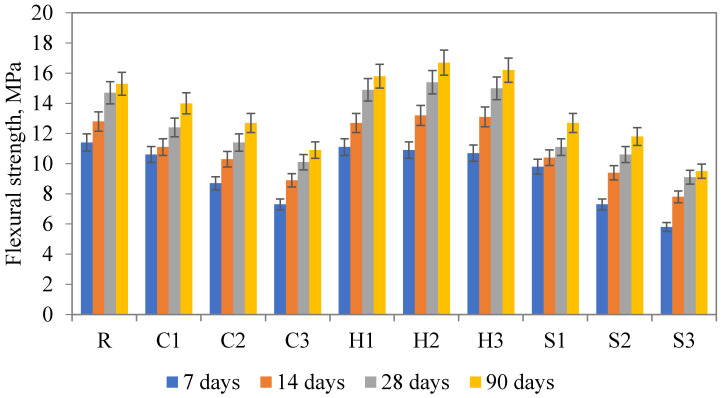
Flexural strength of studied materials.

The results of the compressive and flexural strength determined at various ages (7, 14, 28, and 90 days) are given in Figure 8 and Figure 9. The revealed findings point to an ambiguous effect on mechanical strength. On the one hand, the compressive strength was dropped for all C and S mixtures when compared to the reference sample. The explanation of the worsening of the mechanical performance, apart from the negative effect on the fresh mixture rheology [46], can be assigned to the possible clumping of SAP particles and substantially increased water dosage. In particular, the negative effect of even small changes in w/c was noted by AzariJafari et al. [47] and Justs et al. [48] for lower levels of w/c, compared to contradictory results described by Beushausen et al. [49] who did not recognize any significant changes in the mechanical strength of the studied mortars with the SAP admixture. On the other hand, the incorporation of Hydropam having the biggest particle diameter resulted in a minor improvement in mechanical strength even for 1 wt.% admixture. The obtained findings comply with the conclusion of Kalinowski et al. [43], who recommended the application of larger SAPs due to benefits associated with limited clumping and improvements in pore structure formation. Substantial contradiction can be found in the research performed by De Meyst et al. [44], who highlighted the importance of the cross-linking density of applied SAPs on the swelling rate, thus the effect on compressive strength. On the other hand, did not reveal any significance of the particle size in the range from 10 to 100 µm. The RILEM recommendation suggests one to avoid using particles larger than 350 µm, however, this suggestion is based on a numerical study only [50].

Observed favorable findings associated with Hydropam application consists probably of a suitable particle size that limits the clumping of particles, chemical composition with a lower swelling rate, and minor impact on the fresh mixture workability. The observed increase in compressive strength (about 6%) complies with limited consumption of the batch water during the mixture preparation, creation of a limited amount of voids (in contrast to the other two SAP types), and consequent effective hydration of unreacted cement particles [31]. The continuous hydration of cement particles was observed almost for all studied mixtures since the strength increase between 28 and 90 days overcame the increase in the same period for the reference mixture. A similar material performance was achieved for the flexural strength, thus the H2 mixture revealed the most favorable performance among others, namely the flexural strength after 90 days of curing reached about 10% improvement compared to the reference sample. The observed beneficial results obtained for H mixtures are in contrast with most of the published papers since the decrease in the compressive strength as a result of SAP incorporation is dominant [14,16,51].

## 4. Conclusions

Within this study, the characterization of the selected SAPs and consequent mortars design was done by the meaning the absorption capacity and consequent effects on the fresh mixture workability in order to contribute to a better understanding of the specifics associated with the application of SAPs in the cement mortars design.

Due to the properties of Creasorb and Hydropam, new methods for the absorptivity evaluation has been proposed, since the classical teabag and filtration methods could cause considerable inaccuracies in the obtained results. The applied methods allow minimizing gel leakage in the case of Hydropam and absorption of an excessive amount of liquid for Creasorb. The highest absorptivity value for all SAPs was observed for the distilled water, the lowest for cement-based solutions. In all tested SAPs absorptivity decreased in the tap water and the sodium chloride solution, but the difference in that factor between those two solutions was rather low. In the fifth minute of the experiment, for water and NaCl solution, the Cablock absorptivity reaches equilibrium values, while for Hydropam it gradually increased with elapsed time. The Cablock absorptivity characteristic was completely different in the case of cement-based solutions, where clear sorption maximum was observed for the 5th minute, followed by the desorption step and weight loss of swelled SAP. The desorption stage and associated weight loss were characterized by a lower rate in comparison to the absorption period.

The increase in solution pH caused a decrease in SAPs absorptivity. Cablock superabsorbent polymer had the highest pH-sensitivity, while Creasorb had the lowest. After 1 h the absorptivity for Cablock (in the examined pH range) decreased about 50 times, while Creasorb changed less than 2 times. To quantitatively describe SAP sorption/desorption characteristics, the correlation of absorptivity as the function of the SAP residence time within the examined solution was introduced.

The main contribution of the submitted paper lies in the description of the dependency between the fresh mixture consistency and applied type of SAP taking into account its particle size. Important findings can be found in the results of the sorption experiments and the sensitivity of studied SAPs on changes in solution pH.

As a major drawback for the utilization of SAPs in concrete design can be seen in a low diameter of SAP particles and consequent rapid swelling. Notwithstanding, the more advanced understanding of the effect of SAP on the functional properties of cement-based materials since the current state-of-the-art does not provide coherent guidelines including the crucial characteristics of suitable SAP. The complex understanding and balancing of the benefits associated with the self-healing capability and limited autogenous shrinkage provided by used SAP need to be balanced with drawbacks such as worsening of fresh mixture workability, excessive extra water dosage, large voids formations, etc. In a nutshell, moderation of SAP swelling velocity and capability represents a challenging task that needs to be resolved to provide SAP material with tailored characteristics for concrete design.

## Figures and Tables

**Table 1:** Initial mixture composition.

Mixture	Cement (kg/m^3^)	w/c	Sand (kg/m^3^)	Superplasticizer (%)	SAP (%)	ew/c (-)
R	450	0.3	1350	0.4	0	0
C1	450	0.3	1350	0.4	0.6	0–0.1
C2	450	0.3	1350	0.4	0.8	0–0.1
C3	450	0.3	1350	0.4	1	0.1–0.15
H1	450	0.3	1350	0.4	0.6	0–0.1
H2	450	0.3	1350	0.4	0.8	0–0.1
H3	450	0.3	1350	0.4	1	0.1–0.15
S1	450	0.3	1350	0.4	0.6	0–0.1
S2	450	0.3	1350	0.4	0.8	0–0.1
S3	450	0.3	1350	0.4	1	0.1–0.15

**Table 2:** ln(*a*) and *b* coefficients depending on used solution–SAP system.

	Solution	SAP Type	ln(*a*)	*b*	R^2^
**S**	Distilled Water	Hydropam	−0.1883	−0.0007	0.9069
	Tap Water	Hydropam	−0.2317	−0.0007	0.963
	0.1M NaCl	Hydropam	−0.213	−0.0008	0.9894
	10 w/c	Hydropam	0.0285	−0.0019	0.9668
	5 w/c	Hydropam	−0.3445	−0.0008	0.9642
	2.5 w/c	Hydropam	−0.3558	−0.0006	0.9248
	Distilled Water	Cablock	−0.2942	−0.0073	0.8831
	Tap Water	Cablock	−0.2524	−0.0072	0.9743
	0.1M NaCl	Cablock	− 0.6031	−0.0063	0.9009
	10 w/c	Cablock	−1.6484	−0.0028	0.1196
	5 w/c	Cablock	−1.7422	−0.0038	0.1729
	2.5 w/c	Cablock	−1.746	−0.0059	0.6307
**D**	10 w/c	Cablock	1.7005	0.0005	0.6019
	5 w/c	Cablock	2.0023	0.0006	0.6193
	2.5 w/c	Cablock	1.7696	0.0005	0.5519

**Table 3:** Optimized mixture composition.

Mixture	Cement (kg/m^3^)	w/c	Sand	Superplasticizer (%)	SAP (%)
R	450	0.3	1350	0.4	0
C1	450	0.34	1350	0.4	0.6
C2	450	0.37	1350	0.4	0.8
C3	450	0.44	1350	0.4	1
H1	450	0.32	1350	0.4	0.6
H2	450	0.34	1350	0.4	0.8
H3	450	0.41	1350	0.4	1
S1	450	0.36	1350	0.4	0.6
S2	450	0.38	1350	0.4	0.8
S3	450	0.46	1350	0.4	1
